# The Epidemiologic, Microbiologic and Clinical Picture of Bacteremia among Febrile Infants and Young Children Managed as Outpatients at the Emergency Room, before and after Initiation of the Routine Anti-Pneumococcal Immunization

**DOI:** 10.3390/ijerph13070723

**Published:** 2016-07-19

**Authors:** Eugene Leibovitz, Nuphar David, Haya Ribitzky-Eisner, Mouner Abo Madegam, Said Abuabed, Gabriel Chodick, Michal Maimon, Yariv Fruchtman

**Affiliations:** 1 Pediatric Emergency Medicine Department, Soroka University Medical Center, Faculty of Health Sciences, Ben-Gurion University, Beer-Sheva 84101, Israel; nuphardavid@gmail.com (N.D.); huyas@hotmail.com (H.R.-E.); abomadeg@post.bgu.ac.il (M.A.M.); saidysr@yahoo.com (S.A.); MichalMaimon@clalit.org.il (M.M.); Yariv@bgu.ac.il (Y.F.); 2 Sackler Faculty of Medicine, Tel-Aviv University, Tel Aviv 6997801, Israel; hodik_g@mac.org.il

**Keywords:** occult bacteremia, bacteremia with focus, *Streptococcus pneumoniae*, blood cultures, leukocytosis, antibiotics

## Abstract

We described the occult bacteremia (OB) and bacteremia with diagnosed focus (BwF) picture among children managed as outpatients at the pediatric emergency room (PER) in southern Israel, before and after the introduction of pneumococcal conjugate vaccines (PCVs) introduction in a retrospective study enrolling all three- to 36-month-old patients with fever >38.0 °C during 2005–2014. Of 511 (0.82% of all febrile patients) true bacteremias, 230 (45%) were managed as outpatients; 96 of 230 (41.7%) had OB and 134 (3.59%) had BwF. OB and BwF rates were 0.22% and 3.02%, respectively. A significant decrease was noted in OB and BwF rates (*p* = 0.0008 and *p* = 0.02, respectively). *S. pneumoniae* (SP, 37.5%), *K. kingae* (11.4%) and *Brucella* spp. (8.7%) were the most common OB pathogens and SP (29.8%), *S. viridans* (13.4%), and *Brucella* spp. (12.7%) were the most common in BwF patients. PCV13 serotypes were not found among the serotypes isolated post-PCV13 introduction. During 2010–2014 there was an increase in non-PCV13 serotype isolation (*p* = 0.005). SP was the main pathogen isolated among patients with pneumonia, acute otitis media (AOM) and periorbital cellulitis (62.5%, 33.3% and 60%, respectively). OB and BwF decreased following the introduction of PCVs and SP was the main pathogen in both conditions. Vaccine-SP serotypes were not isolated in OB after PCV13 introduction and non-vaccine serotypes increased significantly.

## 1. Introduction

While the epidemiological, clinical and microbiological picture of bloodstream infections (BSIs) has been frequently described among adult patients, information is lacking about this phenomenon among children, and if it exists, it refers mainly to nosocomial infections and not to children examined and discharged from the pediatric emergency room (PER) [1,2].

Children with BSI managed at a PER visit can be divided into two groups: BSI associated with a diagnosed infectious focus (bacteremia with focus, BwF), and BSI without a focus (including clinically suspected sepsis or occult bacteremia, OB). Of them, patients with OB and a considerable amount of those with BwF may be managed as outpatients. Obtaining blood cultures is a standard procedure for the diagnosis of bacteremia and blood cultures are performed frequently at PER. However, while the indication for obtaining blood cultures in patients with clinical suspicion of sepsis or meningitis is obvious, this indication in children who appear well and are suspected of being at risk for OB is less well defined, being related particularly to specific age and temperature cut-offs. Furthermore, obtaining blood cultures may be a common practice in certain focal infections (such as pneumonia or urinary tract infections) and much less common in others (such as acute otitis media, tonsillitis or cellulitis).

OB may be present under the syndrome of fever without a source, particularly in infants/young children between the ages of three to 36 months [3,4,5,6,7,8]. It was shown that around 1.5%–11% (mean 4.3% in patients with a fever >39 °C) of children with fever without a source may develop an infectious focus or sepsis as a result of OB [3,4]. Children with a fever >39 °C, leukocytosis >15,000/mm^3^ or <5000/mm^3^, bandemia and a high erythrocyte sedimentation rate or C-reactive protein (CRP) are considered at high risk of OB [3,4,5,6]. Furthermore, pneumonia may be diagnosed by X-ray in 20%–30% of the febrile children less than five years without any suggestive clinical findings, but with leukocytosis >20,000/mL [9]. Traditional protocols (according to the Baraff criteria) required that young children with fever and >15,000 leukocytes/mm^3^ should receive antibiotic treatment (oral amoxicillin or intramuscular/intravenous ceftriaxone) at their discharge from PER and continue the therapy until receiving the results of the blood cultures [3,4,5,6].

Today, the use of the anti–*H. influenzae* type b vaccine, together with the relatively new use of the vaccination against *S*. *pneumoniae* (SP, PCVs, pneumococcal conjugate vaccines), has completely changed the picture of OB among three- to 36-month-old children and made it rare [10,11,12,13,14,15,16]. The introduction of PCVs was associated with a major decrease in the incidence of invasive pneumococcal infections in children [13,14,15,17,18,19]. In a study analyzing the impact of PCV7 on pneumococcal bacteremia diagnosed in children aged 0–18 years at the PER of a tertiary-care children’s hospital in Philadelphia during 1998–2009, the annual proportion of disease caused by PCV7 serotypes decreased from 82.7% during the pre-PCV7 period to 19.5% peri- and post-PCV7 [20].

In Israel, PCV7 was introduced to the national immunization plan in July 2009, and was replaced by PCV13 in November 2010. There is no data in southern Israel, and, in fact, in Israel, regarding the pathogen rate and distribution as well as the epidemiological and clinical picture of BSI/OB/BwF among febrile three to 36-month-old children examined and diagnosed at the PER. Furthermore, no data is available on the possible changes in the incidence of BSI/OB/BwF and in the microbiological picture of febrile children/young children following the introduction of routine immunization with PVCs.

The main objectives of the present study were to characterize and compare the epidemiological, clinical and microbiological picture of OB and of BwF among febrile infants/children examined and managed as outpatients at a PER in southern Israel, before and after the introduction of PCVs.

## 2. Patients and Methods

We completed a retrospective cohort study enrolling all three- to 36-month-old infants/young children diagnosed during 2005–2014 at the PER of the Soroka Medical Center in Be’er Sheva, Israel, with BSI with an infectious focus or with OB and who were then discharged to their homes. All of the data was extracted from the computerized medical charts and microbiological laboratories’ data of these children. OB was defined as the presence of a pathogenic bacteremia in the blood of a well-appearing febrile child in the absence of an identifiable focus of infection [3,4]. For OB, we analyzed the medical records of all patients with a discharge diagnosis of:

1. Fever without source;
2. Suspected occult bacteremia;
3. Fever only.

Patients with immunodeficiencies, malignancies and presence of long-term vascular catheters were excluded. In the process of patient evaluation and diagnosis of the “well-appearing” status, we considered various elements, such as the documentation of vital signs, skin color and exanthems, behavioral status and assessment of the hydration state [3,4]. We recorded the blood pressure and obtained pulse oximetry. The temperature was measured rectally. Children were examined for the presence of petechiae. The diagnosis of fever without source was considered if no source of infection was apparent after a thorough examination in a nontoxic infant or child without any significant underlying illness [3,4]. 

The study was conducted in accordance with the Declaration of Helsinki. Approval to perform this study was obtained from the institutional review board (protocols no. 0138-12-SOR and 0197-16-SOR).

The Soroka University Medical Center is the only general hospital in southern Israel and provides primary and tertiary health services to the entire population of southern Israel. It served a population of >800,000 inhabitants in 2012, of them >250,000 were children <18 years of age. The PER of the hospital accepts around 36,000 visits/year. 

In southern Israel, two pediatric populations live side by side: Jewish children, largely urban with a lifestyle comparable to Western populations, and Bedouin children, formerly composed of desert nomads, and now in transition to a Western lifestyle. Hospitalization rates due to respiratory and gastrointestinal infections in general, and invasive pneumococcal diseases particularly, are more prevalent among Bedouin children [14,21,22,23].

Today, all the infants in Israel have been immunized with three doses of the *H. influenzae* type b vaccine (at the age of two, four and six months) since 1994. PCV7 was available from 2006 on the private market. Since 1 July 2009, all the infants in Israel have received routinely two doses of the Prevenar vaccine (Prevenar13 since 15 November 2010, at the age of two and four months). A booster dose with both *H. influenzae* and PCV13 vaccines is administered at the age of one year. The vaccine coverage in the Israeli pediatric population is >90%.

### 2.1. Study Population

The study population included all the infants/young children three to 36 months old diagnosed in the PER with fever without or with a specific infectious source, discharged form PER and with a positive blood culture (representing OB or BwF with a true pathogen reported following the visit). The status of the PCV immunization of the enrolled patients was not available or not reliably documented from the patients’ medical charts.

### 2.2. Treatment Protocol

The treatment protocol in children with suspicion of OB in the PER required performing blood cultures in every patient with a temperature >38.0 °C. The decision to treat OB with antibiotic drugs belonged to the treating physician based on presence/absence of a peripheral white blood cell count >15,000 cells/mm^3^. In general, even when leukocytosis >15,000 cells/mm^3^ was present, there was still a recommendation of refraining from antibiotic treatment if the patient’s family was considered compliant and reliable and an appropriate follow-up could be assured. When an antibiotic treatment was prescribed, the protocol recommended oral amoxicillin (80 mg/kg/day in two daily doses) or intramuscular/intravenous ceftriaxone (50 mg/kg once daily), until the results of blood cultures became available.

For BwF, blood cultures were obtained in all children diagnosed with pneumonia and urinary tract infection. For the other diagnosed focal infections (except tonsillitis), blood cultures were performed when a temperature >38.0 °C was recorded.

### 2.3. Study Conduct

The patients were identified based on the diagnosis at discharge from the PER together with positive microbiology diagnoses provided by the microbiology laboratories of the hospital. We documented the patients’ age, sex and ethnicity, background diseases, clinical presentation, complete blood count, microbiological agents responsible for OB and BwF and their susceptibility to antibiotics and antibiotic treatment at discharge. 

### 2.4. Microbiology

*S. pneumoniae* isolates (non-meningeal) were considered susceptible to penicillin (by parenteral administration) if the minimal inhibitory concentration (MIC) values were ≤2 µg/mL, intermediate if penicillin MIC values were 4 µg/mL and resistant if MIC values were ≥8.0 µg/mL. Ceftriaxone intermediate resistance was defined by MIC values between 0.5 and 1.0 µg/mL, and high resistance by MIC values >2.0 µg/mL [24].

The following bacterial species were considered contaminants: *Staphylococcus* coagulase negative, *Corynebacterium* spp. and other diphteroids, α-coagulase streptococci, *Propionibacterium acnes* and *Micrococcus* spp., if recovered in otherwise healthy patients. When doubt existed in respect to the potential pathogenicity of one of the species isolated, the final decision was reached after further analysis of the specific patient files and additional consultation with infectious disease specialists. In patients with BwF, the pathogen isolated in the blood culture did not necessarily represent the true etiologic agent considered responsible for the respective infectious focus.

### 2.5. Statistical Analysis

Data were computerized and analyzed with the SPSS 19.0 package (SPSS Inc., Chicago, IL, USA). ANOVA and *t*-test were used to compare continuous variables. The chi-square test and chi-square test for trend were used for parametric data distribution in the univariate analysis of contingency tables, and trends over time in OB and BwF rates. *p*-values < 0.05 were considered statistically significant. The year 2009 was not included in some calculations because it was inconclusive in terms of vaccination completion (vaccination was initiated on 1 July 2009).

## 3. Results

During the study period, 169,406 children aged three to 36 months visited the PER and 62,252 of them were diagnosed with fever >38 °C (Table 1). Of the 511 of 62,252 (0.82%) patients with true bacteremia reported, 230 (45.0%) were discharged after the PER visit; 96 of 230 (41.70%) were diagnosed with OB and 134 (59.30%) with BwF.

**Figure 1:**
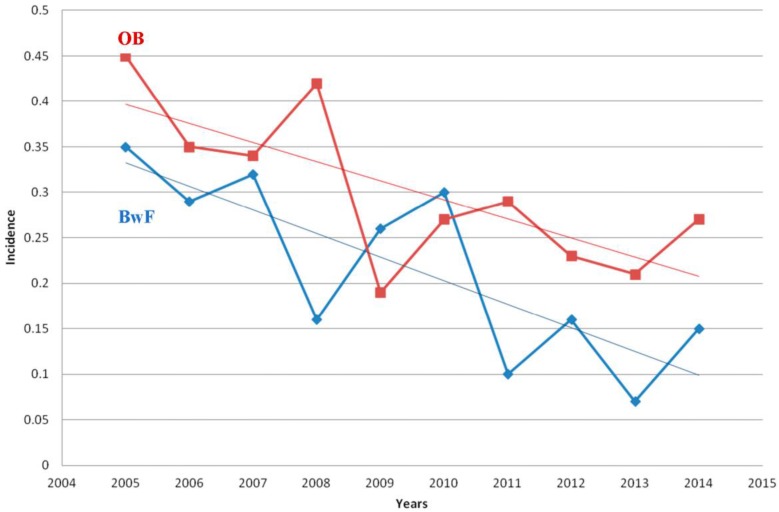
Epidemiologic trends of OB (occult bacteremia) and BwF (bacteremia with focus) during the study years. OB—red line; BwF—blue line. Incidence expressed as number of cases of OB and BwF/all febrile infants and children discharged from the PER.

Overall rates of OB and BwF (among all febrile infants and children discharged from the PER) during the study period were 0.22% and 0.30%, respectively (Table 1). More OB cases were documented before the introduction of PCVs (2005–2008) compared with the period after their introduction (2010–2014): 53 of 19,160 (0.27%) vs. 31 of 19,870 (0.15%), *p* = 0.01. More BwF cases were documented before the introduction of PCVs compared to the period after the introduction of PCVs: 75 of 19,160 (0.39%) compared to 51 of 19,870 (0.26%), *p* = 0.019. During the whole study period, a significant decrease in the OB rates was noted (*p* = 0.0008, chi square for linear trends in proportions); likewise, a significant decrease was documented in BwF rates (*p* = 0.02) (Figure 1).

Of the 230 discharged patients, 120 (52.20%) children were of Jewish origin and 110 (47.80%) of Bedouin origin; there were 130 (56.30%) males and 100 (43.50%) females, and 44.3% were three to 11 months of age, 38.30% were 12–23 months of age and 17.40% were 24–36 months of age. No differences were documented between these age groups in the distribution of the OB and BwF cases. In addition, 183 of 230 (79.60%) patients did not have any background diseases.

Overall, SP was the most common pathogen (76/230, 33.0%)), followed by *S. viridans* (10.90%), *K. kingae* (10.0%), *Brucella* spp. (8.70%), and *E. coli* and *Salmonella* spp. (4.34% each) (Table 2). Among patients with OB, SP was the main pathogen (37.50%), followed by *K. kingae* (11.40%), *Brucella* spp. (8.70%), and *E. coli* and *Salmonella* spp. (7.30% each). Among patients with BwF, SP was isolated in 29.8% of the patients, with no difference in its representation in this group compared with the OB group (*p* = 0.2); *S. viridans* was the second most common pathogen (13.40%), *Brucella* spp. the third (12.70%, a significantly higher representation compared with patients with OB, *p* = 0.01) and *K. kingae* the fourth pathogen (8.95%, with no difference in its representation compared with the OB patients). *Enterobacteriaceae* spp. represented 9.60% (fourth in descending order) of all 230 discharged patients, with no difference in their percentages between OB and BwF patients.

No significant changes were recorded in the distribution of SP or any of the other pathogens isolated in OB or in BwF during the study years. When comparing the pre-PCVs period (2005–2008) with the post-PCVs period (2010–2014) and excluding the year 2009 (not indicative in terms of vaccination completion, because PCV7 was introduced only on July 2009) from the calculations , no differences were recorded in the percentages of the five most common pathogens isolated (in decreasing order) among all BSI cases (SP, *K. kingae*, *Brucella* spp., *Salmonella* spp. and *S. aureus*): 35.70% vs. 26.80% of all isolates, 10.10% vs. 9.70%, 6.2% vs. 12.20%, 4.70% vs. 4.90% and 3.90% vs. 2.40%, *p* = 0.18, 0.93, 0.13, 1.0 and 0.7, respectively. Similarly, no significant changes were recorded in the distribution of the five most frequently isolated pathogens isolated in OB (SP, *K. kingae*, *Salmonella* spp., *S. aureus* and *Brucella* spp.) or in BwF (SP, *Brucella* spp., *K. kingae*, *E. coli* and *Salmonella* spp.) between the pre and post-PCVs time periods.

SP was isolated in 18 of 47 (38.30%) of the Jewish children and in 18 of 49 (36.70%) of the Bedouin children with OB (*p* = 0.9). *Brucella* spp. was not isolated among Jewish patients with OB vs. three cases (6.10%) among Bedouin patients with OB (*p* = 0.24). *K. kingae* was isolated in nine of 47 (19.10%) of the Jewish children with OB, vs. two of 49 (4.10%) of the Bedouin children (*p* = 0.02). *Brucella* spp. was not isolated in Jewish patients with BwF, but was isolated in 17 of 61 (27.9%) of the Bedouin children with BwF (*p* < 0.001). *K. kingae* was isolated among 11 of 73 (15.10%) of the Jewish patients with BwF, vs. one of 61 (1.60%) of the Bedouin patients with BwF (*p* = 0.007). 

The average peripheral blood white blood cell (WBC) count among all patients was 16,632.3 ± 8124.1/mm^3^, with no difference between the children with OB (15953.9 ± 7889.5) and those with BwF (1711.8 ± 8281.8, *p* = 0.1); >15,000 WBC/mm^3^ were found in 1.470% of the patients, with no difference between the children with OB and those with BwF (42.6% and 49.6%, *p* = 0.09).

No differences were recorded in the representation of SP in cases with OB between the period before (2005–2008) and that after (2010–2014) the introduction of PCVs (nine of 34 cases, 35.20%, vs. 11 of 31, 35.50%, *p* = 0.97). The representation of SP in BwF in the period after the introduction of PCVs was lower than in the period before their introduction (11 of 51, 21.60% vs. 27 of 75, 36.0%, *p* = 0.08). No difference was shown in the distribution of the other most common pathogen recorded in OB and BwF (*K. kingae*, *S*. *aureus*, *Brucella* spp., *E. coli* and *Salmonella* spp.) between the two time periods.

Further, 4.460% and 8.540% of the children with OB did not receive any antibiotic treatment for this condition before and after the vaccine introduction, respectively (*p* = 0.45). No differences in the therapeutic choice (amoxicillin or ceftriaxone) for OB were documented between the two study periods (amoxicillin administered during 2005–2008 in 16 of 54 cases, 29.60% vs. seven of 31, 22.60%, during 2010–2014, *p* = 0.77; ceftriaxone administered during 2005–2008 in seven of 54 cases, 12.90% vs. seven of 31, 22.60%, during 2010–2014, *p* = 0.45). 

No mortality was recorded in both OB and BwF patients.

A total of 76 SP cases were reported, 36 in OB and 40 in BwF. Overall, the most commonly isolated serotypes were 19A (11.80%), 6B (11.80%), 1 (10.50%), 9V (7.90%) and 14 (7.90%). The most common serotypes isolated in OB were 19A (22.20%), 1 (8.30%), 6B (8.30%), 9V (8.30%), 12F (5.50%), 14 (5.50%) and 19F (5.50%) (Table 3). The most common serotypes isolated in BwF were 6B (15.0%), 1 (12.50%), 14 (10.0%), 9V (7.50%), 5 (5.0%), 19F (5.0%) and 23F (5.0%). Serotype 19A was isolated more frequently in OB compared with BwF (nine of 36, 25.0% vs. one of 40, 2.50%, *p* = 0.01); no differences were recorded between OB and BwF in the representation of the other pneumococcal serotypes. During the study years, PCV7 covered 33.30% of the SP-OB isolates and 42.50% of the SP-BwF isolates (12 of 36 vs. 17 of 40, *p* = 0.4). PCV13 covered 69.40% of the SP isolates in OB compared with 70.0% in BwF (25 of 36 vs. 28 of 40, *p* = 0.05). The representation of non-PCV13 serotypes was similar in OB and BwF (30.60% vs. 30.00%, *p* = 0.95).

Overall, PCV13 serotypes were not found among the serotypes isolated in OB after the introduction of PCV13 (Table 4). All four SP serotypes isolated in OB during 2011–2014 were not included in PCV13. During 2011–2014 there was a decrease in PCV13 serotype isolation and an increase in the non-PCV13 serotype isolation (*p* = 0.05 and *p* = 0.005, respectively). For BwF, eight of nine (88.90%) of the SP serotypes isolated during 2011–2014 were not included in PCV13.

Further, 46.40% (25 of 54) and 54.80% (17 of 31) of the patients with OB during 2005–2008 and 2010–2014, respectively, did not receive any antibiotics with their discharge from the PER. No differences were recorded in the percentages of OB patients treated with amoxicillin or ceftriaxone between these two time periods (29.60% vs. 22.6 for amoxicillin, *p* = 0.77 and 12.90% vs. 22.6% for ceftriaxone, *p* = 0.25).

All SP isolates of OB were susceptible to penicillin and ceftriaxone. There were 25 (69.40%) isolates with a MIC to penicillin ≤0.06 µg/mL, seven with a MIC between 0.25 and 1.0 µg/mL and four isolates with a MIC of 2 µg/mL (serotypes 14, 19A, 19F and 23F). One OB isolate had a MIC to ceftriaxone of 0.25 µg/mL (serotype 19A). 

Among the BwF isolates, all were sensitive to penicillin; 30 (75.0%) had a MIC to penicillin ≤0.06 µg/mL, four (10.0%) had a MIC between 0.25 and 1.0 µg/mL and six (15.0%) isolates had a MIC to penicillin of 2 µg/mL (four isolates with serotype 1, 19A, 19F and 23 F and two isolates with serotype 6B). Thirty-seven (92.50%) isolates had a MIC to ceftriaxone ≤0.25 µg/mL and three had a MIC of 1 µg/mL (two serotypes 6B and one serotype 19A). 

In addition, 124 of the patients with BwF had one diagnosis and 10 had two diagnoses (two focuses). The most common infectious focuses were pneumonia (28.0%), acute otitis media (23.0%), gastroenteritis (17.50%), brucellosis (10.0%), arthritis/limping (2.70%) and urinary tract infection (2.70%) (Table 5). SP was the main pathogen isolated among patients with pneumonia, acute otitis media (AOM) and periorbital cellulitis (62.50%, 33.30% and 60.0% of all the cases, respectively). Of the 17 cases of *Brucella* spp. (BwF), two had frank arthritis and the other 15 had, in addition to bacteremia, focal osteoarticular involvement (arthralgia) and general signs and symptoms (weakness, fever, weight loss, lymphadenopathy or sweating) without arthritis. All 20 patients with *Brucella* spp. bacteremia had *B. melitensis* isolated from the blood cultures and had an initial positive agglutination serologic test for the pathogen.

## 4. Discussion

We described in the present study the epidemiologic, microbiologic and clinical aspects of BSI occurring in febrile infants/young children aged three to 36 months examined at the PER and managed as outpatient due to their healthy-looking condition. We analyzed these BSI as cases of OB and cases of BwF in the only tertiary-care hospital in southern Israel and were able to present our data as population-based. We presented our data as pre (2005–2008) and post (2010–2014) introduction of PCVs in the routine immunization program in Israel.

We showed in this study that: (1) The average rates of OB and BwF were low during the study period and both decreased following the introduction of PCVs; (2) SP was the main pathogen isolated among patients with OB and BwF, without differences in its distribution between OB and BwF; (3) *K. kingae* was isolated mainly among Jewish children, while *Brucella* spp. was isolated only among Bedouin children; (4) There were no significant changes in the distribution of any of the pathogens isolated in OB or BwF during the study period; (5) PCV13 covered 69.50% of the OB cases caused by SP and 70.0% of BwF cases, without any changes in the serotype distribution in these two conditions before and after the vaccine introduction; (6) Vaccine SP serotypes were not recorded after PCV13 introduction and non-vaccine SP serotypes increased significantly after PCV13 introduction; (7) Most of the SP-OB and SP-BwF isolates were susceptible to penicillin and ceftriaxone; (8) The main focuses associated with BwF were pneumonia, AOM and acute gastroenteritis and SP was the main pathogen isolated among patients with pneumonia, AOM and periorbital cellulitis. 

The introduction of the PCVs in pediatric immunization programs all around the world was accompanied by a rapid decrease in the prevalence of pneumococcal invasive diseases and, as expected, a decrease in the OB rates in various medical centers in the USA and western Europe to <1% [15,16,25,26,27,28,29,30]. Ben Shimol et al. [17], in a study on invasive pneumococcal disease (IPD) epidemiology in children under five years in Israel, following the introduction of PCV7 in July 2009, found that in 2009 and 2010, PCV7 serotypes and IPD incidences decreased by 43.0% and 81.0%, respectively, compared to the pre-PCV7 period 2003–2007. The same group [18] reported on the sequential introduction of PCV7/PCV13 on IPD and demonstrated an impressive decrease (95.0%) in the rates of disease caused by PCV7+6A in the PCV13 period. The IPD caused by the additional PCV13 serotypes (1, 3, 5, 7F and 19A) increased initially by 47.0% but subsequently decreased by 79.0% (overall 70.0% reduction during 2004–2013). On the other hand, a two-fold increase in the incidence of IPD caused by non-PCV13 serotypes was reported [18]. In southern Israel, the overall OB rate recorded in children aged three to 36 months at PER during 2005–2012 was 0.22%, with a significant decrease during the post-PCV period (2010–2012) compared with the pre-PCV period (2005–2009). SP (39.30%) was the most frequently isolated pathogen in OB and an increase in the non-PCV13 serotype recovery was shown following the introduction of the PCV13 vaccine in 2010 [30]. 

Today, in the PCV era, the pathogens shown to cause bacteremia in young children are *Neisseria meningitidis*, *Escherichia coli*, *Staphylococcus aureus*, group A *Streptococcus* and *Salmonella* spp., and also non-vaccine serotypes of *S*. *pneumoniae* [15]. At the PER level, SP was reported as the leading cause of bacteremia in a prospective multicenter study enrolling healthy patients aged 0–18 years from 15 PERs during 2011–2013 [31]. Among the 711 studied patients with a positive blood culture, 46.90% were under one year old and 80.50% were under five years old. The most common final diagnosis was OB (28.60%); 109 patients (15.20%) met the criteria for sepsis and the most common focal infections associated with bacteremia were urinary tract infections and pneumonia (18.0% and 14.0%, respectively). One hundred and seventy (23.90%) patients were initially managed as outpatients. Overall, the most frequently isolated bacterial species was SP (27.30%), followed by *E. coli* and *S. aureus* (20.60% and 12.60%, respectively). Our retrospective study had some similarities with the previously mentioned one, but concentrated only on the patients managed as outpatients and did not look after bacteremias in admitted patients. We showed a significant decrease in the prevalence of both OB and BwF at our PER during the study period and also a significant decrease in the number of cases caused by the PCV13-SP serotypes during the short period of time following the introduction of this vaccine. This decrease was impressive and was characterized by the complete disappearance of the PCV13-SP serotypes as the etiologic agents of both OB and BwF. The fact that the overall rates of pneumococcal OB and BwF did not reach significantly decreased rates during the study period was related, most probably, to the lack of efficacy of the initially introduced PCV7 against some important SP serotypes (particularly 19A) and also due to the serotype replacement phenomenon with non-vaccine SP serotypes. Indeed, we showed a significant increase in the rates of non-PCV13 serotypes isolated during the post-PCV13 introduction, accompanying the major decrease in PCV13-SP serotypes, similarly to previously reported data [15,17,18,20]. 

We showed in the present study that leukocytosis >15,000/mm^3^ was present at the PER examination in <50% of the cases of OB and BwF (42.60% and 49.60%, respectively). These finding are similar to previously published data showing a poor predictive value of leukocytosis in the diagnosis of OB [15,16,31,32]. In a retrospective case series of all blood cultures obtained between 1998 and 2003 in northern California outpatient clinics and emergency departments from previously healthy children three to 36 months old, Herz et al. showed that leukocytosis >15,000/mm^3^ is a poor predictor of bacteremia in febrile toddlers (positive predictive value of 1.50%) and in children routinely immunized with PCV7 [15]. Hernandez-Bou [33] showed recently, in 591 children aged three to 36 months vaccinated with PCV13 in a tertiary-care medical center in Spain, that an elevated band count was the best predictor of the uncommon (six patients only, 1.0%) cases of OB that were diagnosed, with 66.70% sensitivity and 93.30% specificity, while the positive predictive values of a WBC count >15,000/mm^3^, C-reactive protein >40 mg/L and procalcitonin >0.5 ng/mL were low. It is clear, today, taking into consideration the low rates of OB in patients vaccinated with PCVs, that use of the white blood cell count alone to guide the empiric use of antibiotics is not indicated anymore, and that new guidelines advocating for a management change are needed in the approach to the previously healthy febrile toddler [7,8,16,20,28,33,34].

*K. kingae* was the second (11.40%) most common pathogen recovered in our series in OB and the fourth in frequency (8.95%) in BwF. In both OB and BwF cases, *K. kingae* was isolated in significantly more Jewish than Bedouin patients. *K. kingae* is considered an important pediatric pathogen today and is responsible for the majority of the osteoarticular infections in children <36 months [35,36,37,38,39,40]. The clinical picture of *K. kingae* infection is of a relatively mild condition and the clinical findings are not specific, and therefore this pathogen should always be considered in the etiology of fever without a source in infants and young children [38,39,40,41,42]. 

*Brucella* spp. was not isolated among Jewish patients with OB or BwF; from the 20 BSI cases caused by this pathogen occurring in Bedouin children only, 17 had BwF and the majority (15 cases) were associated with focal osteoarticular involvement and general signs and symptoms (weakness, fever, weight loss, lymphadenopathy or sweating) without frank arthritis. In Israel, the disease is caused by *B. melitensis* only and is endemic in the southern region of the country, being almost exclusively diagnosed among the Bedouin population living in close proximity to unvaccinated sheep and goats and consuming unpasteurized dairy products from these animals [43,44]. In children, an unproportionally high incidence of 16 cases per 100,000 was reported during 2005–2011 in Bedouin children <18 years [43]. In a recent study from our medical center, Fruchtman et al. [44] showed that bacteremia was diagnosed in >50.0% of the children with brucellosis (22.6% of them under four years). Fever at diagnosis, however, was recorded in only 20% of the patients, making the probability that the disease will present as OB or BwF relatively remote [44]. 

This study has some limitations, the main one deriving from the retrospective method of data collection, which, in a natural way, may have influenced some of the patients and disease. The lack of exact information on the previous PCV immunization status of the patients in this study represented another limitation in the final analysis of the enrolled patients' characteristics. The relatively short period of time (2010–2014) available for the examination of the changes in OB rates following the PCV13 introduction might have been insufficient in order to draw definitive conclusions from this study. Although we demonstrated, in an unequivocal way, a significant decrease in the overall OB and SP-OB rates in children immunized with the PCVs, a longer follow-up might be needed in order to better understand the PCV13 impact, together with an additional analysis of the replacement phenomenon of the vaccine pneumococcal serotypes by non-vaccine serotypes.

## 5. Conclusions

The average rates of OB and BwF were low during the study period and both decreased following the introduction of PCVs. SP was the main pathogen isolated among patients with OB and BwF. PCV13 coverage was similar for SP isolated in OB and BwF without any changes in the serotype distribution of these two conditions before and after vaccine introduction. SP-PCV13 serotypes were not recorded after PCV13 introduction and non-vaccine SP serotypes increased significantly after PCV13 introduction. We established the distribution and etiology of the BwF cases and reported that SP was the main pathogen isolated among patients with bacteremia associated with pneumonia, AOM and periorbital cellulitis.

## Figures and Tables

**Table 1:** Occult bacteremia (OB) and bacteremia with focus (BwF) among children with fever >38 °C discharged from the PER during 2005–2014.

Year	No. Children with Fever Discharged from PER	OB		BwF	
		N	(%)	N	(%)
**2005**	4895	17	0.35	22	0.45
**2006**	4809	14	0.29	17	0.35
**2007**	4941	16	0.32	17	0.34
**2008**	4515	7	0.16	19	0.42
**2009**	4217	11	0.26	8	0.19
**2010**	4370	13	0.30	12	0.27
**2011**	4088	4	0.10	12	0.29
**2012**	3840	6	0.16	9	0.23
**2013**	4209	3	0.07	9	0.21
**2014**	3363	5	15.0	9	27.0
**Total**	**43,247**	**96**		**134**	
**Mean ± SD**	**4324.7 ± 496.5**	**9.6 ± 5.21**	**0.216**	**13.4 ± 4.97**	**0.302**

**Table 2:** Bacteremia at PER: pathogen distribution (in decreasing frequency).

	Total N (%)	OB N (%)	BwF N (%)	*p* Value
***Streptococcus pneumoniae***	76 (33.0)	36 (37.50)	40 (29.8)	0.2
***Kingella kingae***	23 (10.0)	11 (11.40)	12 (8.95)	0.5
***Streptococcus viridans* spp.**	25 (10.87)	7 (7.30)	18 (13.40)	0.14
***Salmonella*** **spp.** *	10 (4.34)	7 (7.30)	3 (2.20)	0.1
***Gram negative*** **bacilli**	8 (3.50)	5 (5.21)	3 (2.20)	
***Acinetobacter*** **spp.**	9 (3.90)	5 (5.21)	4 (3.0)	
***Staphylococcus aureus***	7 (3.0)	5 (5.21)	2 (1.50)	
***Neisseria*** spp. **	5 (2.17)	3 (3.12)	2 (1.50)	
***Brucella*** **spp.**	20 (8.70)	3 (3.12)	17 (12.70)	0.01
*Escherichia coli* *	10 (4.34)	2 (2.10)	8 (6.0)	0.20
***Enterococcus*** **spp.**	2 (0.87)	2 (2.10)	0 (0)	
***Pseudomonas*** **spp.**	9 (3.90)	2 (2.10)	7 (5.20)	0.31
***Enterobacter cloacae*** *	1 (0.42)	1 (1)	0 (0)	
***Gram positive*** **cocci**	2 (0.87)	2 (2.10)	0 (0)	
***Haemophilus influenzae*** non-typable	6 (2.60)	1 (1)	5 (3.70)	0.40
***Klebsiella pneumoniae*** *	1 (0.43)	1 (1)	0 (0)	
***Pantoea agglomerans***	1 (0.43)	1 (1)	0 (0)	
***Pasteurella multocida***	1 (0.43)	1 (1)	0 (0)	
***Streptococcus pyogenes***	5 (2.17)	1 (1)	4 (3.0)	
**OTHER**	9 (4.90)	0 (0)	9 (6.70)	
**Total**	**230 (100%)**	**96 (100%)**	**134 (100%)**	

* *Enterobacteriaceae* spp. = 22 (9.60%) patients, of them 11 (11.40%) OB and 11 (8.20%) BwF (*p* = 0.41); ** Of the five *Neisseria* spp. isolated, three were identified as *N. meningitidis*; the other two could not be specified as *N. meningitidis*, but were considered true pathogens based on the clinical and laboratory picture.

**Table 3:** Serotype distribution of *S. pneumoniae* isolated during the study years—comparison between OB and BwF.

Serotype	Total	OB	BwF	*p* Value
	*N* = 76	*N* = 36	*N* = 40	
19A	9	8	1	0.01
1	8	3	5	0.7
5	2	-	2	
6B	9	3	6	0.49
9V	6	3	3	1.0
19F	4	2	2	1.0
12F	3	2	1	0.6
14	6	2	4	0.67
23F	4	2	2	1.0
3	1	1	-	
6A	4	1	3	0.62
6C	1	1	-	
10A	1	1	-	
15	1	-	1	
15A	1	-	1	
15B	2	-	2	
15B/C	3	1	2	1.0
18A	1	1	-	
21	1	-	1	
24F	1	1	-	
28A	1	1	-	
33A	1	-	1	
33F	2	1	1	
34	1	-	1	
35B	2	1	1	
46	1	1	-	
Total PCV7	29 (38.20%)	12 (33.30%)	17 (42.50%)	
Additional six seroytpes	24 (31.60%)	13 (36.10%)	11 (27.50%)	
Total PCV13	53 (69.70%)	25 (69.50%)	28 (70.0%)	
Non-PCV13 serotypes	23 (30.30%)	11 (30.50%)	12 (30.0%)	
Total	76 (100%)	36 (100.0%)	40 (100%)	

Notes: Prevenar7 (introduced in July 2009) = 4, 6B, 9V, 14, 18C, 19F, 23F; Prevenar13 (introduced in November 2010) = 1,3,4,5,6A,6B,7F,9V,14,18C,19A,19F,23F.

**Table 4:** Serotype distribution of pneumococcal OB: comparison between 2005–2010 and 2011–2014 (PCV13 and non-PCV13 serotypes).

Serotype	2005–2010	2011–2014	*p* Value
**OB (*N* = 36)**	***N*** **= 32**	***N*** **= 4**	
PCV13 isolates	25	0	0.05
Non-PCV13	7	4	0.005
	(6C, 10A, 12F, 18A, 24F, 28A, 46)	(12F, 15B/C, 33F, 35B)	
**BwF (*N* = 40)**	***N*** **= 31**	***N*** **= 9**	
PCV13 isolates	27	1	
Non-PCV13 isolates	4	8	
	(15A, 33A, 33F, 34)	(12F, 15, 15B-2, 15B/C-2, 21, 35B)	

**Table 5:** BwF: specific diagnoses/focuses at discharge from the ER (in descending frequency) *.

Diagnosis	Total BwF Cases	Most Common Pathogen (No. of Cases Caused by the Pathogen, %)
Pneumonia	40	*S. pneumoniae* (25, 62.50 %)
		*S. viridans* (8, 20.0%)
		*H. influenzae* non-typable (3, 7.50%)
Acute Otitis Media	33	*S. pneumoniae* (11, 33.30%)
		*K. kingae* (5, 15.20%)
Acute Gastroenteritis	25	*S. viridans* (7, 28.0%)
		Gram negative bacilli (3, 12.0%)
		*Salmonella* spp. (3, 12.0%)
Brucellosis **	15	*-*
Periorbital Cellulitis	5	*S. pneumoniae* (3, 60.0%)
Urinary Tract Infection	4	*E. coli* (3, 75.0%)
Limping/Arthritis	4	*Brucella* spp. (2, 50.0%)
		*K. kingae* (2, 50.0%)
Acute Tonsillitis	3	
Aphtous Stomatitis	2	*S. aureus* (1, 50.0%)
Cellulitis	1	
Acute Otitis Externa	1	S. aureus (1)
Fever Convulsion	1	
Other	6	
Total	143	

* The pathogens isolated in the blood cultures did not necessarily represent the pathogens responsible for the infectious focus; ** Positive blood cultures for *Brucella* spp. accompanied by focal osteoarticular involvement and general signs and symptoms without frank arthritis.
